# Characterization of metabolism-associated molecular patterns in prostate cancer

**DOI:** 10.1186/s12894-023-01275-w

**Published:** 2023-06-06

**Authors:** Bowei Yang, Yongming Jiang, Jun Yang, Wenbo Zhou, Tongxin Yang, Rongchang Zhang, Jinming Xu, Haixiang Guo

**Affiliations:** Department of Urology, The Second Affiliated Hospital of Kunming Medical University, Kunming, China

**Keywords:** Prostate cancer, Metabolism, Molecular subclusters, Tumor environment, Prognosis

## Abstract

**Background:**

Metabolism is a hallmark of cancer and it involves in resistance to antitumor treatment. Therefore, the purposes of this study are to classify metabolism-related molecular pattern and to explore the molecular and tumor microenvironment characteristics for prognosis predicting in prostate cancer.

**Methods:**

The mRNA expression profiles and the corresponding clinical information for prostate cancer patients from TCGA, cBioPortal, and GEO databases. Samples were classified using unsupervised non-negative matrix factorization (NMF) clustering based on differentially expressed metabolism-related genes (MAGs). The characteristics of disease-free survival (DFS), clinicopathological characteristics, pathways, TME, immune cell infiltration, response to immunotherapy, and sensitivity to chemotherapy between subclusters were explored. A prognostic signature was constructed by LASSO cox regression analysis based on differentially expressed MAGs and followed by the development for prognostic prediction.

**Results:**

A total of 76 MAGs between prostate cancer samples and non-tumorous samples were found, then 489 patients were divided into two metabolism-related subclusters for prostate cancer. The significant differences in clinical characteristics (age, T/N stage, Gleason) and DFS between two subclusters. Cluster 1 was associated with cell cycle and metabolism-related pathways, and epithelial-mesenchymal transition (EMT), etc., involved in cluster 2. Moreover, lower ESTIMATE/immune/stromal scores, lower expression of HLAs and immune checkpoint-related genes, and lower half-maximal inhibitory concentration (IC50) values in cluster 1 compared with cluster 2. The 10 MAG signature was identified and constructed a risk model for DFS predicting. The patients with high-risk scores showed poorer DFS. The area under the curve (AUC) values for 1-, 3-, 5-year DFS were 0.744, 0.731, 0.735 in TCGA-PRAD dataset, and 0.668, 0.712, 0.809 in GSE70768 dataset, 0.763, 0.802, 0.772 in GSE70769 dataset. Besides, risk score and Gleason score were identified as independent factors for DFS predicting, and the AUC values of risk score and Gleason score were respectively 0.743 and 0.738. The nomogram showed a favorable performance in DFS predicting.

**Conclusion:**

Our data identified two metabolism-related molecular subclusters for prostate cancer that were distinctly characterized in prostate cancer. Metabolism-related risk profiles were also constructed for prognostic prediction.

**Supplementary Information:**

The online version contains supplementary material available at 10.1186/s12894-023-01275-w.

## Introduction

Prostate cancer (PC) is most malignancy in men, with a globally estimated of approximately 1.4 million new cases and 375,000 deaths in 2020 [1]. Although current research has shown that factors such as increasing age, ethnicity, family history of this malignancy, certain genetic mutations, lifestyle and environmental factors are strongly associated with the development of prostate cancer, knowledge of its etiology is still limited [2–5]. Commonly and traditionally, tissues biopsy remains the standard for the diagnosis of suspected prostate cancer, mainly the Gleason classification [6]. The improved risk stratification [7, 8], new image technology [9], new molecular biomarkers [10] increase the precise of diagnosis for men with prostate cancer. Two biomarkers, prostatic acid phosphatase and prostate-specific antigen (PSA) was utilized for screening patient and PSA has gradually replaced prostatic acid phosphatase due to its high sensitivity [11]. However, PSA is specific to the diseases occurred in prostate, but not correlated with the Gleason score, and therefore disconnected with prostate cancer [11, 12]. Therefore, recent studies focus on developing novel biomarkers with improved characteristics in prostate cancer to combined with PSA for clinical decision- making. Nowadays, three primary options, including expectant management, surgery, and radiation, are chosen for localized prostate cancer. Expectant management is a safe and preferred approach for men with less-aggressive prostate cancer [13], surgery and radiation remain be curative treatments for man with more significant cancer [14]. However, the treatment option of surgery and radiation causes the adverse effects, such as urinary symptoms, sexual dysfunctions, and recurrence, affect the quality of prostate cancer patients’ life [15, 16]. Androgen deprivation therapy continues to be the first-line treatment for men with metastatic prostate cancer, but emerges toxicant effect [17]. Chemotherapy and immunotherapy become to be the efficiency treatments to extend survival of prostate cancer [18, 19]. However, metastatic prostate cancer remains incurable.

Metabolic reprogramming becomes a hallmark of solid cancer, and closely related to the tumor development and progress [20, 21]. Metabolic reprogramming generates the necessary nutrients under the nutrient-poor environment and then to support cell viability and build new biomass [22]. Alterations in gene expression, cellular differentiation, and tumor microenvironment (TME) through metabolic reprogramming in intracellular and extracellular during the processes of cancer-associated metabolic reprograming [23]. Typically, the metabolic changes associated with cancer involve reprogramming of glucose, fatty acid, and amino acid, and nucleotide metabolism [24]. Glucose, fatty acid, and amino acid, nucleotide metabolism are the main sources of nutrients for energy supplement and macromolecular synthesis, and constituent of three core metabolic pathways, such as anabolic, catabolic, and waste producing, and mediate biological processes, for example, glycolysis, tricarboxylic acid (TCA) intermediated oxidative phosphorylation, glycogenolysis, lipogenesis, and ureal cycle [25]. Increasing evidences have indicated that metabolic reprogramming exerts critical role in carcinogenesis, progression, treatment, and prognosis of prostate cancer [26, 27]. In the normal prostate tissue, the citrate-orientated metabolic process existed, which may indicate the unique metabolic properties showed in prostate cancer [25]. In primary prostate cancer, tumor enhanced oxidative phosphorylation but limited increase of glycolysis, which was a characteristic of advanced castrate resistant prostate cancer [21, 25]. Also, lipogenesis in the form of fatty acid synthesis [28, 29] and amino acid metabolism plays a crucial role in prostate cancer progression [30]. Guro et al.[31] found that metabolic profiling, especially spermine and citrate content can used to distinct the aggressive from indolent prostate cancer. Thus, investigations of metabolism are important to understand carcinogenesis and cancer developing, and to provide novel insight for efficiency diagnosis and treatments.

Taken together, the purposes of this study are exploring the significance of MAGs in prostate cancer and constructing metabolism-related gene signatures for survival prediction. In the present study, prostate cancer patients were classified into two metabolic-related subclusters based on differentially expressed metabolism related genes (DE-MAGs). The molecular characteristics, tumor microenvironment characteristics, and responses to therapy in subclusters were analyzed. Besides, we also constructed and validated the prognostic gene signature and predictive nomogram based on glucose, fatty acid, and amino acid metabolism MAGs.

## Methods

### Data collection and processing

Transcriptome expression profiles and corresponding clinical data for 489 prostate cancer tissues and 52 non-tumor tissues were obtained from the TCGA-PARD dataset in the TCGA (https://portal.gdc.cancer.gov/) and the cBioPortal database (https://www.cbioportal.org/). Clinical data contained age, pathological stages, Gleason score, prostate-specific antigen (PSA) value, and disease-free survival (DFS). The TCGA-PARD dataset was used as the training set in this study. Moreover, mRNA expression files and corresponding clinical information of GSE70768 and GSE70769 datasets, including age, pathological stages, PSA value, Gleason score, and biochemical relapse (BCR) survival time, were obtained from GEO (https://www.ncbi.nlm.nih.gov/gds). GSE70768 dataset included 199 prostate cancer samples and 111 of them with BCR information (https://www.ncbi.nlm.nih.gov/geo/query/acc.cgi?acc=GSE70768). GSE70769 dataset included 94 prostate cancer samples and 92 of them with BCR information (https://www.ncbi.nlm.nih.gov/geo/query/acc.cgi). GSE70768 and GSE70769 datasets were performed by Illumina HumanHT-12 V4.0 expression beadchip and were used as the testing sets. A total of 948 MAGs were collected from c2.cp.kegg.v7.4.symbols.gmt which was downloaded from Molecular Signatures Database (MSigDB, https://www.gsea-msigdb.org/gsea/msigdb/), the search used the keywords “metabolism”.

### Screening the differentially expressed MAGs

In the present study, differentially expressed genes (DEGs) between prostate cancer tissues and non-tumor tissues were screened using Limma package in R script with the criteria of absolute log2 (fold change, FC) > 1 and adjust *P* value < 0.05, and the results visualized using ggplot package in R script. Then, the differentially expressed metabolism-associated genes (MAGs) were obtained by intersecting the DEGs and MAGs. And the differentially expressed MAGs were visualized using pheatmap package in R script.

### Unsupervised clustering of prostate cancer

Based on the differentially expressed MAGs, 489 prostate cancer samples from TCGA-PARD dataset were classified into different molecular subclusters using unsupervised non-negative matrix factorization (NMF) clustering via NMF R package. The optimal number of clusters was determined by k value at which cophenetic correlation coefficient began to decline. Then, t-distributed stochastic neighbor embedding (t-SNE) were performed to verify the classification performance using the mRNA expression data of DE-MAGs. Kaplan–Meier (KM) DFS curves were drawn using survival R package to validate the correlation between prognosis and classification. The differentially expressed MAGs between molecular subclusters were shown using pheatmap package in R.

### Estimation of the immune cell infiltration

Estimate package in R script was performed to evaluate the EISTTIMATE score, immune score, and stromal score of each sample, with differences between molecular subgroups subsequently detected by the Wilcoxon rank sum test. Besides, single-sample gene set expression analysis (ssGSEA) was conducted based on the mRNA expression data to estimate the immune infiltration by calculating the enrichment score of each gene in a special immune cell marker gene set [32]. SsGSEA was performed using GSVA package in R script, and the discrimination of immune infiltration between molecular subclusters was determined by Wilcoxon rank sum test.

### Gene set expression analysis (GSEA) and gene set variation analysis (GSVA)

The Kyoto Encyclopedia of Genes and Genomes (KEGG) pathway gene set [33–35] (c2.cp.kegg.v7.4.symbols.gmt) and 50 human cancer hallmark gene set (h.all.v7.4.symbols.gmt) were downloaded from MSigDB (https://www.gsea-msigdb.org/gsea/msigdb/). Then, GSEA was performed using the GSEA software (version 4.2.2) to explore the potential molecular mechanism between molecular subclusters. In addition, GSVA was carried out using the GSVA R package to estimate the score of above certain pathways and signatures based on the mRNA expression data. And the distinctions between subclusters were detected by the Wilcoxon rank-sum test. To explore the potential molecular characteristics of TME between the two subclusters, GSVA was performed using GSVA R package between two subclusters. The annotated gene set list, c2.cp.kegg.v7.4.symbols.gmt, and TME related gene set (such as CD8 T effector, DNA damage repair, EMT, Pan-F-TBRS, and nucleotide excision repair genes), were selected as the reference gene set from MSigDB and previous article [36].

### Expression of human leukocyte antigens (HLAs) and immune checkpoint-related genes

HLAs and immune checkpoint-related genes serve an important roles in immune function and were associated with immunotherapeutic sensitivity. Therefore, the different expression of HLAs and immune checkpoint-related genes between subclusters was explored by the Wilcoxon rank-sum test.

### Prediction of the immunotherapeutic response and sensitivity to chemotherapy

The Tumor Immune Dysfunction and Exclusion (TIDE) score was calculated to estimate the likelihood of response to immunotherapy based on the TIDE database (http://tide.dfci.harvard.edu/). And the different TIDE score between subclusters was detected by the Wilcoxon rank-sum test. Besides, Submap mapping was used to investigate the response of anti-CTLA4/PD1 immunotherapy. Considering the distinction in chemotherapeutic sensitivity in prostate cancer patients, the IC50 values of 138 antitumor drugs in Genomics of Drug Sensitivity in Cancer (GDSC, https://www.cancerrxgene.org/) were calculated by ridge regression using pRRophetic package in R script.

### Identification and validation of risk signature and construction of a risk model

Univariate cox analysis was performed to identify the prognosis-related differentially expressed MAGs in the TCGA-PARD dataset. The differentially expressed MAGs associated with DFS were subsequently incorporated into the least absolute shrinkage and selection operator (LASSO) regression cox analysis to construct a metabolism-related risk signature for prognosis. To validate the prognostic value of the risk signature in training (TCGA-PARD) and testing datasets (GSE70768 and GSE70769). The risk score of each sample was calculated according to the following formula, risk score = $\sum_{i=1}^{n}coef\left(genei\right)*expr(genei)$, coef represents the risk coefficient of each gene, and expr represents the expression of each gene. The patients were stratified into high- and low-risk groups based on the median value of the risk score. KM curves were drawn to compare the differences in DFS between high- and low-risk score groups. Moreover, the AUC values of ROC curves for 1-, 3-, and 5-year survival was assessed using the survival ROC package in R script. The differences in clinical characteristics (age, pathological stages, PSA, and Gleason score) between high- and low-risk groups also were compared using the Wilcoxon rank-sum test.

### Estimation of the independent risk factors

The clinical characteristics (age, pathological stages, PSA, and Gleason score) and risk score were subsequently integrated into univariate and multivariate cox analyses to identify the independent risk factors for prostate cancer. Forest plots were constructed to show the independence of the independent risk score. ROC curves for each factor were assessed using the survival ROC package in R.

### Developing a nomogram

The independent risk factors (risk score and Gleason score) which were obtained from univariate and multivariate cox analyses were incorporated into a nomogram to predict the DFS using rms package in R script. The score of each variable was calculated, and then all scores were added up to predict the probability of the outcome of each patient. The higher total score indicated the lower survival rate of each patient. The predictive efficacy of the nomogram was evaluated using calibration curves.

## Results

### Identification of differentially expressed MAGs

**Fig. 1:**
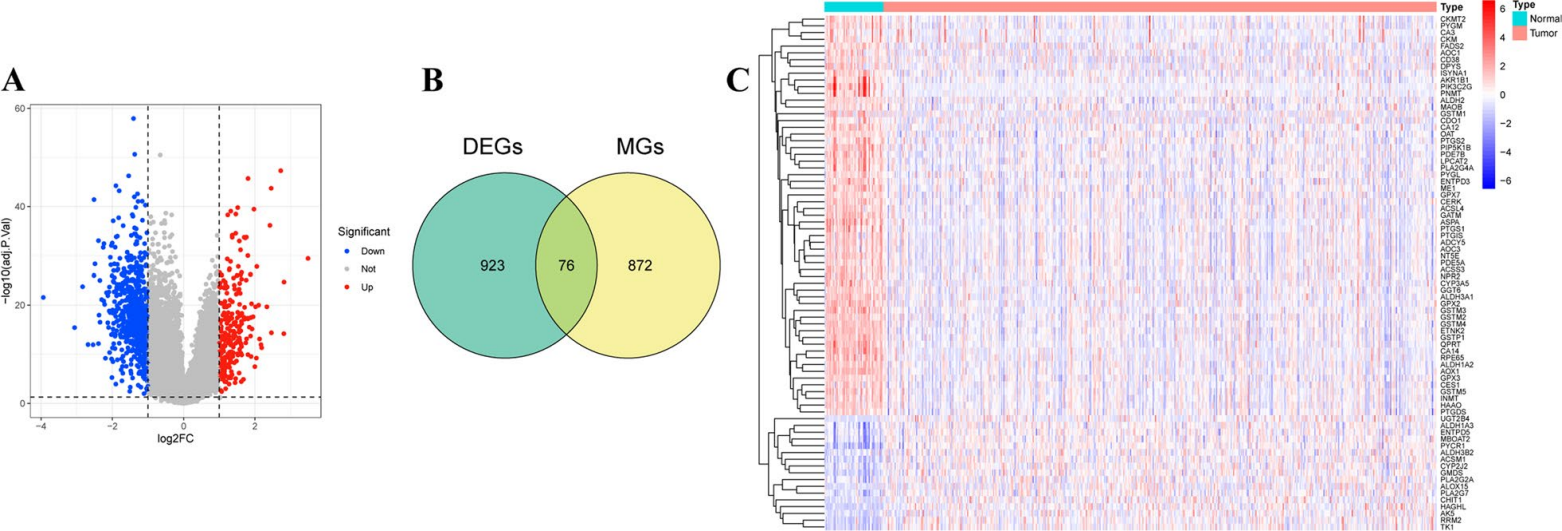
Identification of differentially expressed MAGs. **A** Volcano plot of differentially expressed genes (DEGs) between prostate cancer samples and non-tumor samples. **B** Venn of the differentially expressed MAGs by intersecting DEGs and MAGs from MSigDB. **C** Heatmap clustering of the differentially expressed MAGs between prostate cancer samples and non-tumor samples

We downloaded mRNA expression data of 489 prostate cancer tissues and 52 non-tumor tissues from TCGA, and a total of 999 DEGs, including 252 up-regulated and 747 down-regulated DEGs, were screened out with the criteria of absolute log2 (FC) > 1 and adjusted *P*-value < 0.05 (Fig. 1A, Additional file 3: Table S1). Then, a total of 76 differentially expressed MAGs were obtained by intersecting 999 DEGs and 948 MAGs (Fig. 1B, Additional file 4: Table S2), which included 17 upregulated and 59 downregulated MAGs (Fig. 1C).

### Developing the metabolism-associated molecular patterns

**Fig. 2:**
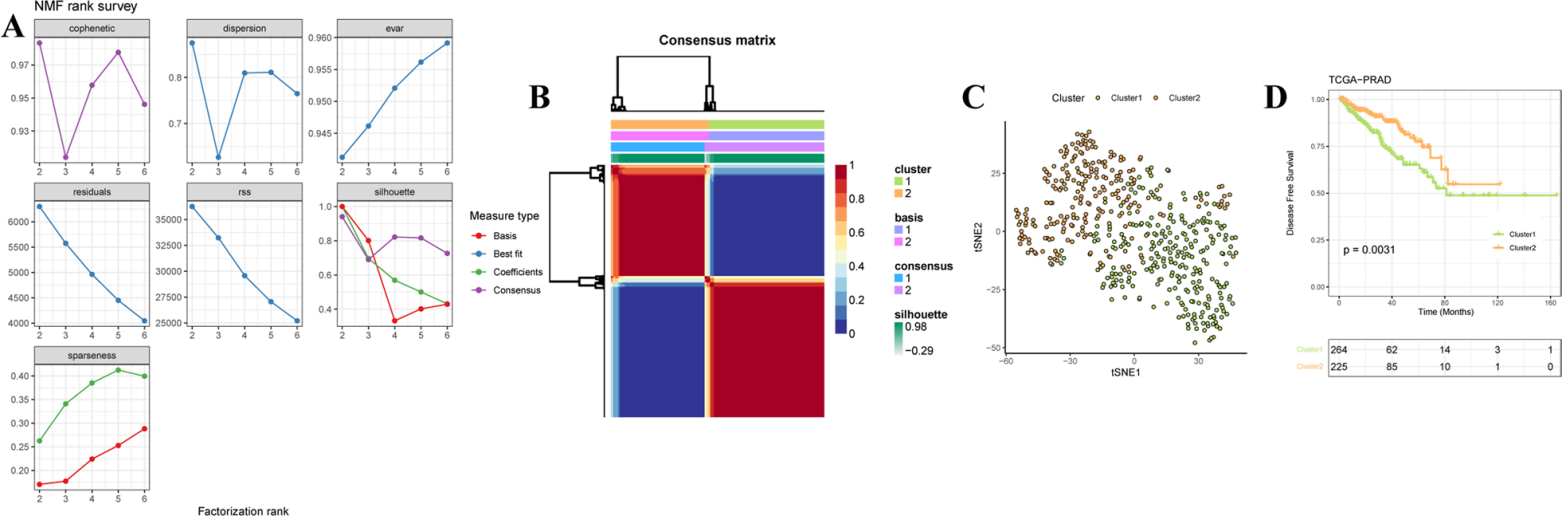
Developing the metabolism-associated molecular patterns. **A** Factorization rank for k = 2–6. **B** Heatmap clustering of the subclusters by NMF clustering with k = 2. **C** T-SNE scatter plots of the distinct two subgroups are shown in accordance with NMF clustering. **D** Kaplan–Meier (KM) DFS curves of the two subclusters in TCGA-PARD cohort

Based on the expression profiles of 76 MAGs, 489 prostate cancer samples were classified into two distinct groups by NMF with the optimal value of k, which was determined by the cophenetic correlation coefficient (Fig. 2A, B, Additional file 1). T-SNE was performed to validate the performance of NMF, resulting supported the classification into two subgroups, including cluster 1 (n = 264) and cluster 2 (n = 225) (Fig. 2C). As shown in Table 1, the significant differences of clinical characteristics were observed between two subgroups, including age (*P* = 0.0434), pathological T stage (*P* < 0.001), pathological N stage (*P* = 0.0013), and Gleason score (*P* < 0.001). KM curve also indicated significantly different DFS between the two subgroups (Fig. 2D, Additional file 1). These data indicated distinct clinical characteristics and prognoses between two metabolism-associated molecular subgroups.

**Table 1:** Clinical characteristics in the metabolic associated molecular subtypes in The Cancer Genome Atlas cohort

Variables	Cluster1 (N = 264)	Cluster2 (N = 225)	*P*-value
Age			
< 60	96 (36.4%)	103 (45.8%)	0.0434
≥ 60	168 (63.6%)	122 (54.2%)	
Pathologic T			
T2	81 (30.7%)	104 (46.2%)	< 0.001
T3	177 (67.0%)	111 (49.3%)	
T4	5 (1.9%)	5 (2.2%)	
NA	1 (0.4%)	5 (2.2%)	
Pathologic N			
N0	176 (66.7%)	164 (72.9%)	0.0013
N1	56 (21.2%)	21 (9.3%)	
NA	32 (12.1%)	40 (17.8%)	
PSA value			
< 4	208 (78.8%)	198 (88.0%)	0.231
≥ 4	17 (6.4%)	9 (4.0%)	
NA	39 (14.8%)	18 (8.0%)	
Gleason score			
< 8	129 (48.9%)	160 (71.1%)	< 0.001
≥ 8	135 (51.1%)	65 (28.9%)	

### Analysis of biofunction between two molecular subclusters

**Fig. 3:**
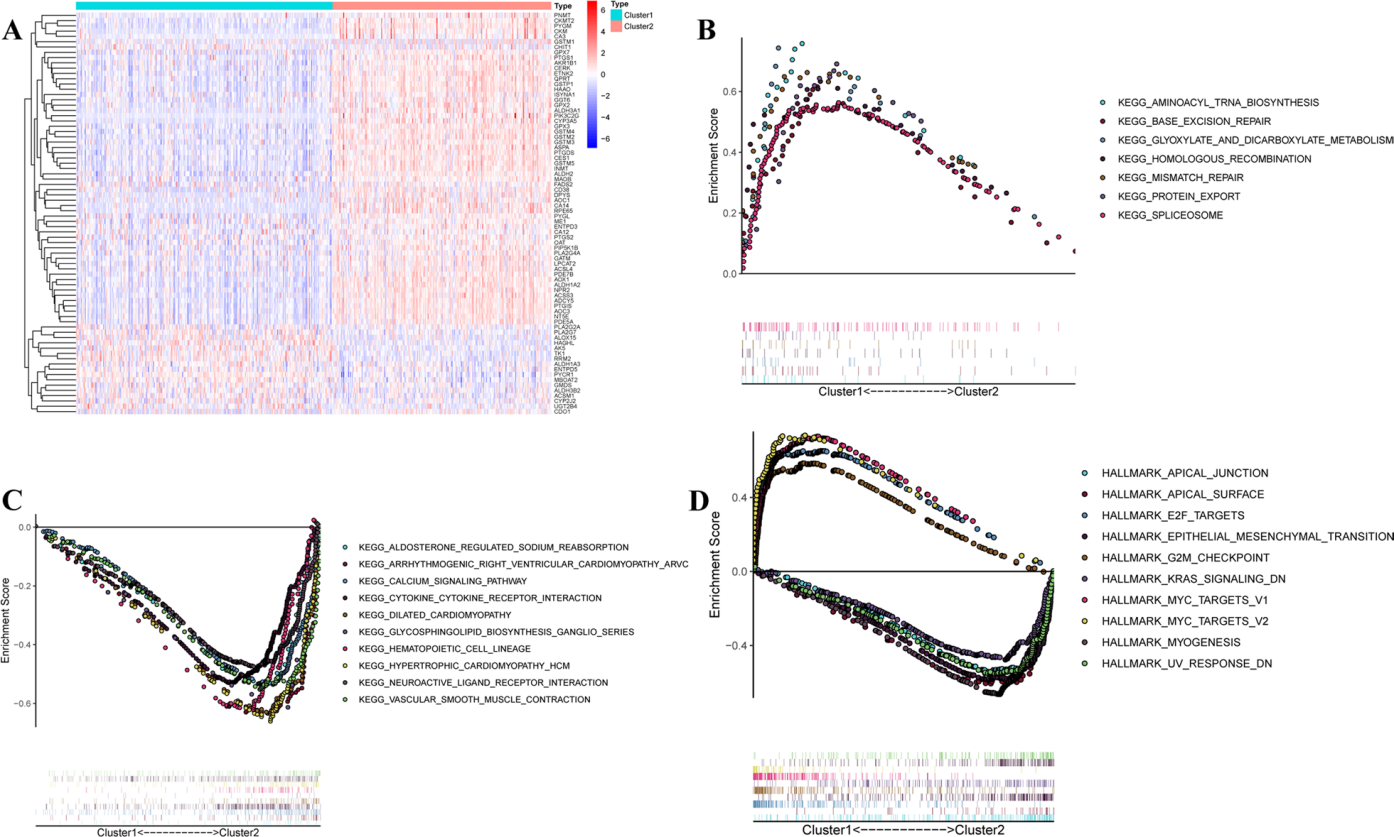
Analysis of biofunction between two molecular subclusters. **A** Heatmap clustering of the differentially expressed MAGs between two subclusters. **B**, **C** GSEA analysis of biological pathways enriched in two subclusters based on KEGG pathways enrichment gene set. **D** GSEA analysis of diseases-related pathways enriched in two subclusters based on 50 human hallmark gene sets

Considering the distinct expression of MAGs between two molecular subgroups (Fig. 3A), we investigated the biofunction pathways enriched in each subgroup using GSEA. And the results showed significant differences in the biofunction enrichment between the two subgroups. Cell cycle and metabolism-related pathways mainly involveed in cluster 1 (Fig. 3B), while two neural regulatory pathways and three cardiac diseases were associated with cluster 2 (Fig. 3C). Besides, aldosterone regulated sodium reabsorption, calcium signaling pathway, cytokine-cytokine receptor interaction, hematopoietic cell lineage, and vascular smooth muscle contraction involved in cluster 2 (Fig. 3C). Additionally, we also investigated the distinct hallmarks of tumors between two subclusters, we found cell cycle-related pathways involved in cluster 1 (Fig. 3D). And myocytes and EMT-related pathways were involved in cluster 2 (Fig. 3D).

### Correlation of the molecular characteristics and subclusters in prostate cancer

**Fig. 4:**
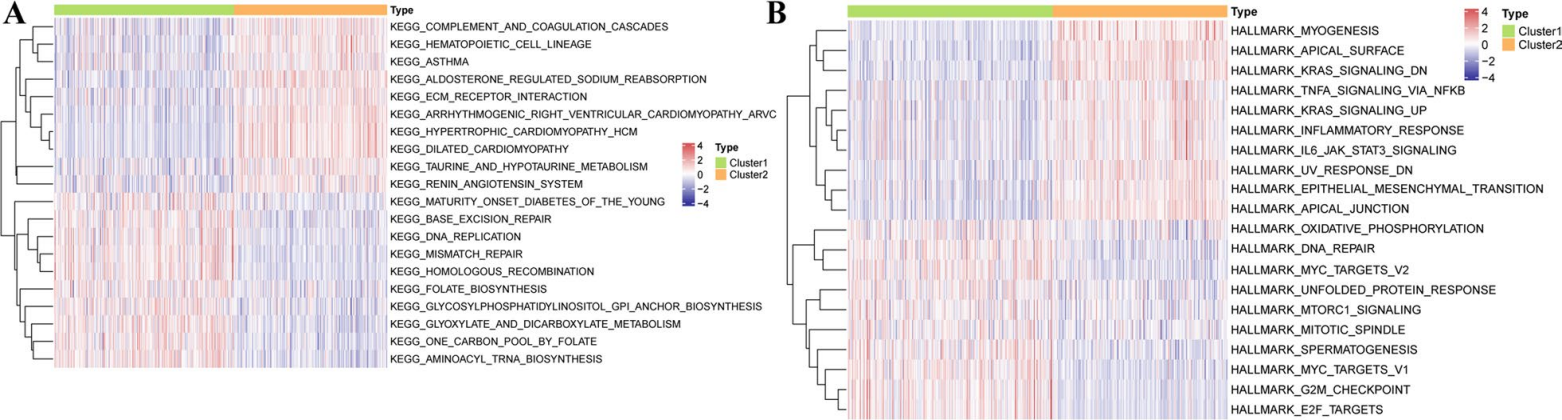
Correlation of the molecular characteristics and subclusters in prostate cancer. **A** GSVA analysis of the different biological pathways enriched in two subclusters based on KEGG pathways enrichment gene set. **B** GSVA analysis of the different diseases-related pathways enriched in two subclusters based on 50 human hallmark gene sets

Considering that the cycle cell, metabolism, cytokine-cytokine receptor interaction, and EMT were involved in prostate cancer, we further explored the different molecular characteristics between two subclusters using GSVA. The results showed that cluster 1 mainly enriched in nucleotide synthesis pathways, folate biosynthesis, glycosylphosphatidylinositol GPI anchor biosynthesis, glyoxylate, and dicarboxylate metabolism (Fig. 4A). And cluster 2 enriched in complement and coagulation cascades, hematopoietic cell lineage, aldosterone-regulated sodium reabsorption, ECM receptor interaction, arrhythmogenic right ventricular cardiomyopathy (ARVC), dilated cardiomyopathy, hypertrophic cardiomyopathy (HCM), taurine and hypotaurine metabolism, renin-angiotensin system (Fig. 4A). Moreover, we further investigated hallmarks of cancer between two subclusters, that resulting in cluster 1 was associated with cell cycle and metabolism associated pathways, (Fig. 4B), and cluster 2 was associated with myogenesis, apical surface, KRAS signaling, TNFα signaling via NF-кB, inflammatory response, IL6-JAK/STAT3 signaling, UV response, epithelial-mesenchymal transition, the apical junction (Fig. 4B).

### Characterization of the TME between two molecular subclusters

**Fig. 5:**
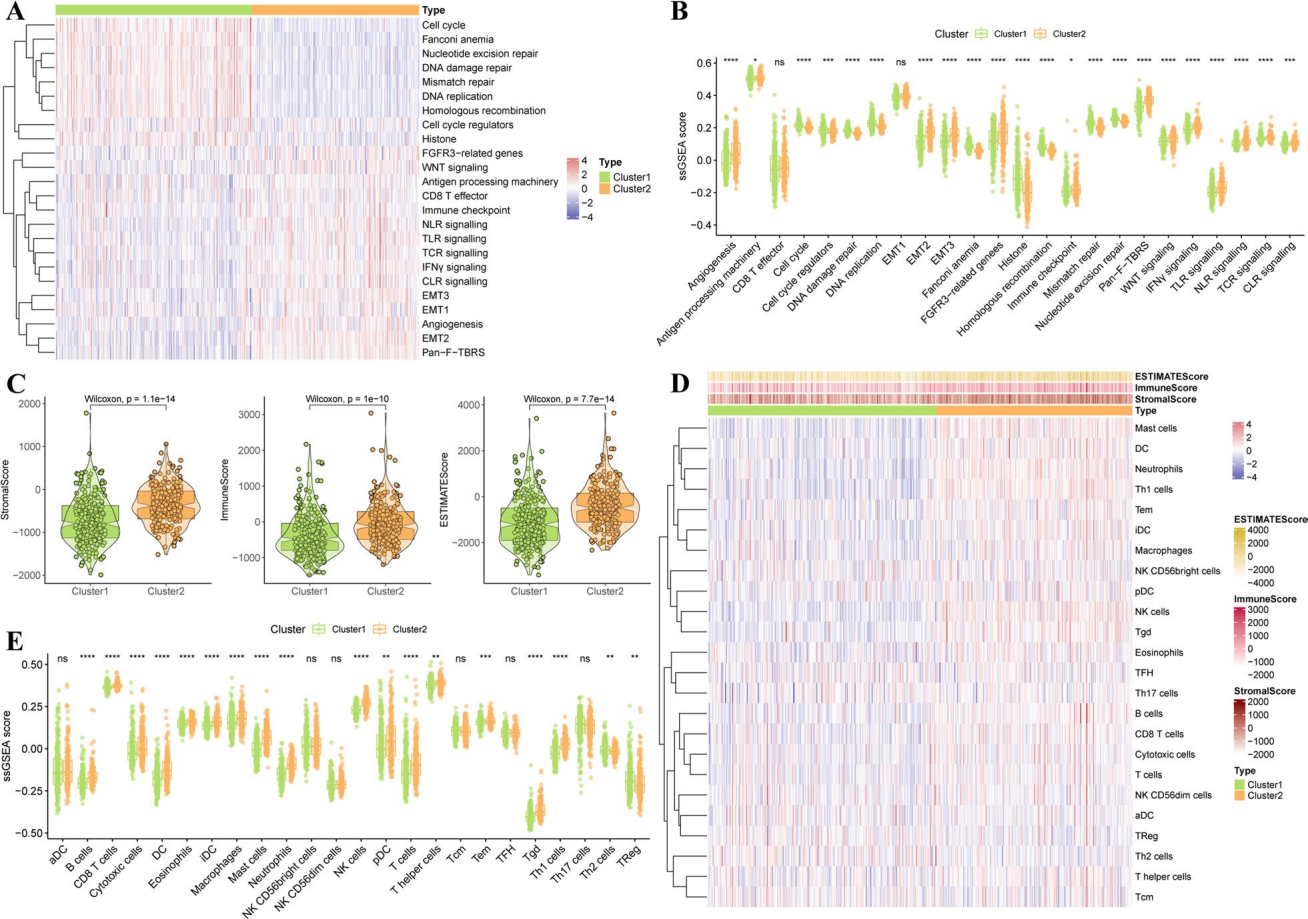
Characterization of TME between two molecular subclusters. **A** GSVA analysis of the different TME-related pathways enriched in two subclusters based on MSigDB and literature. **B** Boxplot of the GSVA enrichment pathways of two subclusters. **C** Violin plot of ESTIMATE score, immune score, and the stromal score of two subclusters. **D** ssGSEA analysis the abundance of immune cells between two subclusters. **E** Violin plot of different immune cell populations between two subclusters using ssGSEA analysis

To better understand the characteristics of TME between distinct molecular subgroups, GSVA was carried out to explore the differences between the two subgroups. As shown in Fig. 5A, B, two subgroups showed the distinct characteristics of TME, cluster 1 was significantly associated with cancer progression-relevant signaling, such as cell cycle, Fanconi anemia, nucleotide excision repair, DNA damage repair, mismatch repair, DNA replication, Homologous recombination, cell cycle regulators, histone. And cluster 2 was associated with stromal and immune activation, such as FGFR3-related genes, WNT signaling, antigen processing machinery, CD8 T effector, immune checkpoint, IFN-γ signaling, NLR signaling, TLR signaling, TCR signaling, CLR signaling, EMT1/2/3, angiogenesis, Pan-F-TBRS (Fig. 5A, B). Subsequently, the immune, stromal and ESITIMATE scores were calculated using ESTIMATE algorithm. Significant differences were exhibited between the two subgroups, that higher stromal scores, immune scores, and ESTIMATE scores in cluster 2 than in cluster 1 (Fig. 5C). We further explored the abundance of 24 immune-related cell types between two subgroups, and the results showed that the different abundance of 18 immune cell populations between two subgroups, B cells, CD8 T cells, cytotoxic cells, dendritic cells (DCs), eosinophils, iDCs, macrophages, mast cells, neutrophils, natural killer (NK) cells, pDC, T cells, T helper cells, Tem, Tgd, Th2 cells were significantly enriched in cluster 2 compared with cluster 1, whereas Th2 and Treg cells increased in cluster 1 than cluster 2 (Fig. 5D, E).

### Response of immunotherapy and targeted therapies in two molecular subclusters

**Fig. 6:**
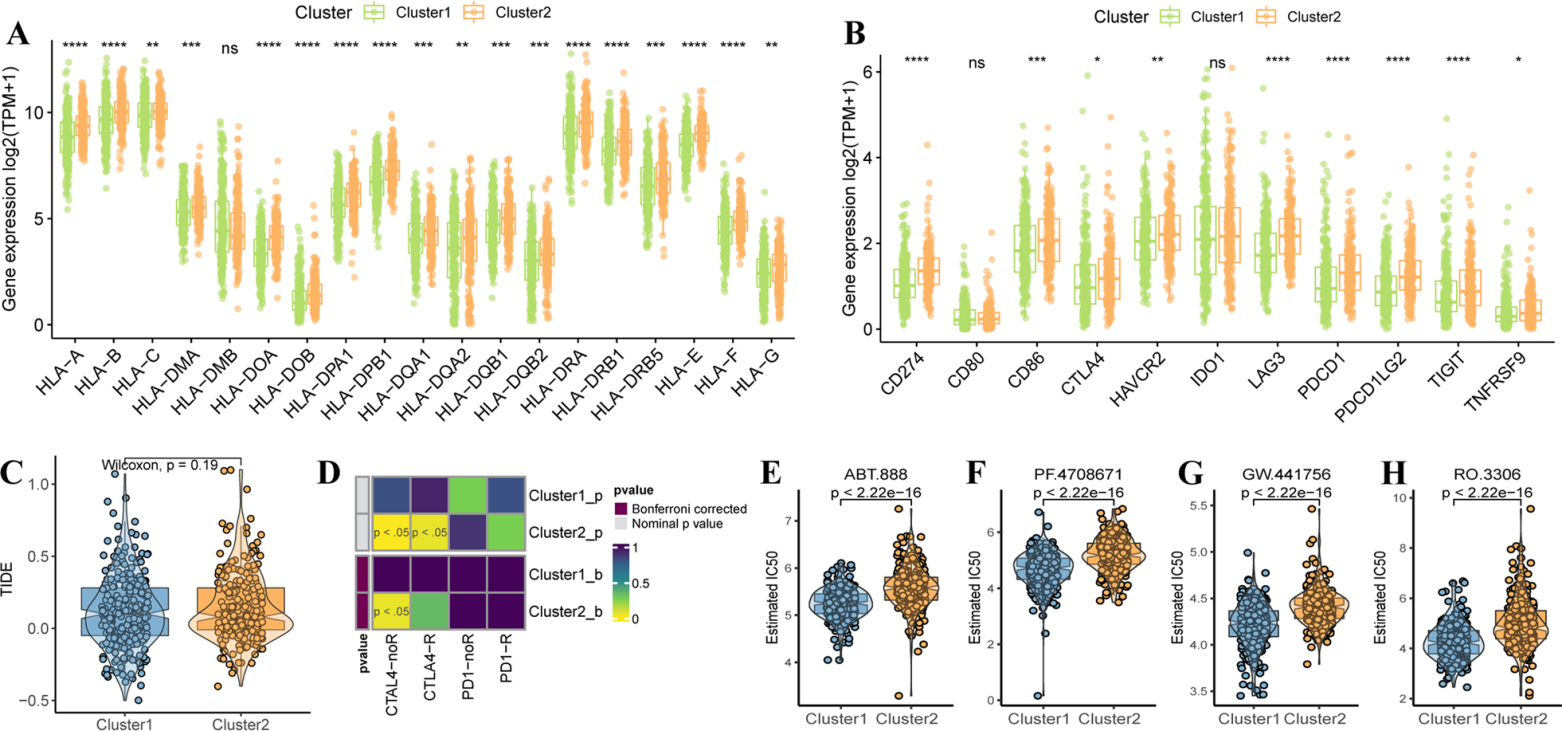
Response of immunotherapy and targeted therapies in two molecular subclusters. **A** Histogram of the differentially expressed HLA family between two subclusters. **B** Histogram of the differentially expressed immune checkpoint-related genes between two subclusters. **C** Violin plot of TIDE prediction score between two subclusters. **D** Submap analysis of the response to anti-CTLA4 and anti-PD-1 treatment of two subclusters. **E**–**H** Violin plot of different IC50 values of ABT.888, PF.4708671, GW441756, RO.3306 between two subclusters

We further investigated the association between subgroups and the expression of HLAs and immune checkpoint related genes, resulting in high expression of HLA-A, -B, -C, -E, -F, -G, -DMA, -DOA, -DOB, -DPA1, -DPB1, -DQA1, -DQA2, -DQB1, -DQB2, -DRA, -DRB1, -DRB5 in cluster 2 compared with cluster 1 (Fig. 6A). And the significant expression of CD274, CD86, CTLA4, HAVCR2, LAG3, PDCD1, PDCD1LG2, TIGIT, and TNFRSF9 in cluster 2 (Fig. 6B). Then, based on the heterogeneity of immune characteristics between two subgroups, we explored the sensitivity of patients who responded to immunotherapy. Although no significant differences in TIDE score between the two subgroups (Fig. 6C). Submap analysis was subsequently used to investigate the response to the anti-PD-1 and CTAL4 treatment, as shown in Fig. 6D, it indicated the response to anti-CTAL4 treatment in cluster 2. Besides, we investigated the chemotherapeutic sensitivity between two subgroups, the patients of cluster 1 showed the sensitive to 31 antitumor drugs than patients in cluster 2 (Additional file 2: Figure S1), and the top 4 chemotherapy drugs were ABT.888, PF.4708671, GW.441756, RO.3306 (Fig. 6E–H).

### Construction and validation of metabolism-associated prognostic signature for prostate cancer

**Fig. 7:**
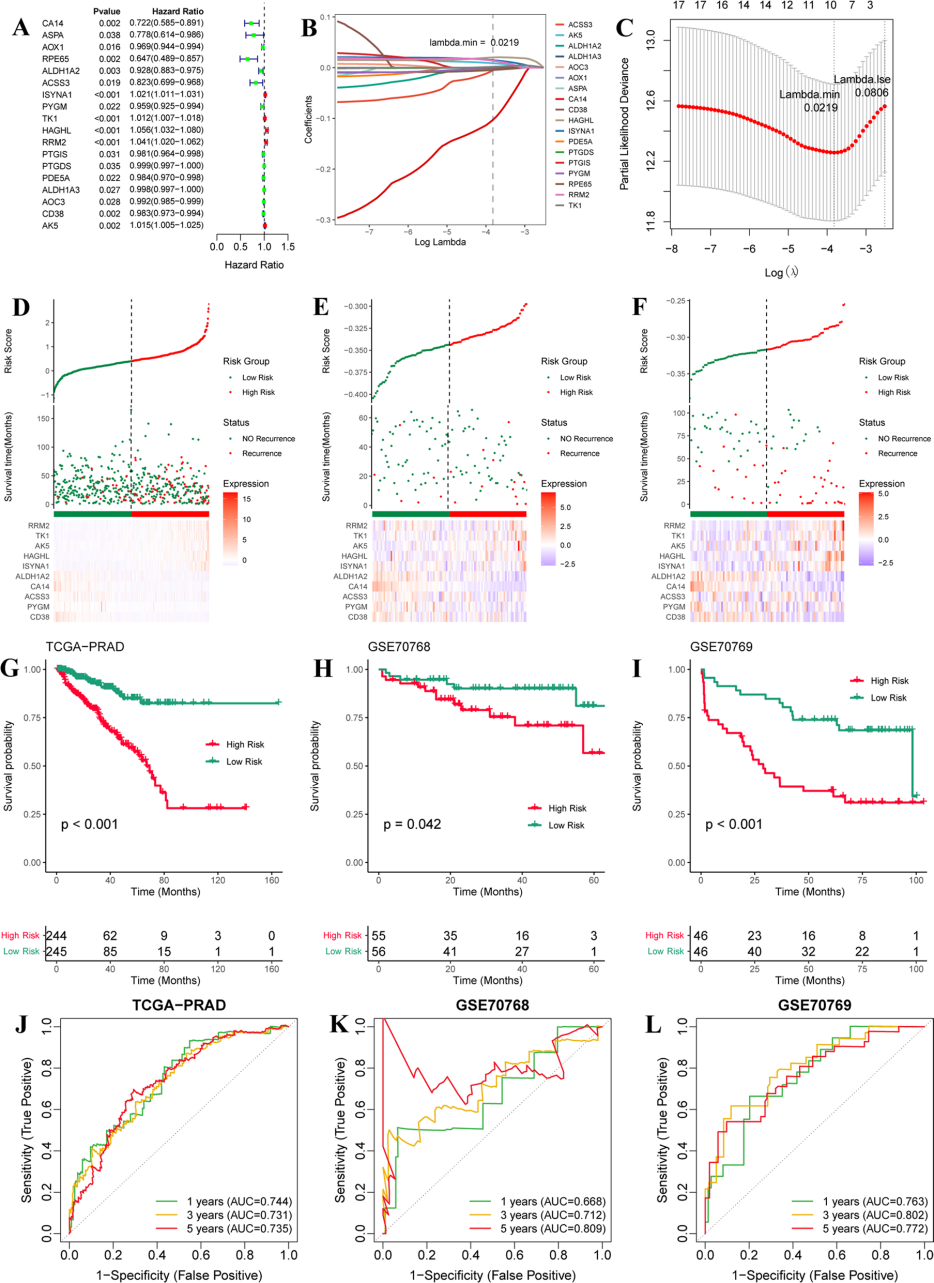
Construction and validation of metabolism-associated prognostic signature for prostate cancer. **A** Forest plot of the DFS-related MAGs based on univariate cox analysis. **B** LASSO coefficient profiles of the selected MAGs at optimal λ (grey line) for metabolism-related clustering. **C** LASSO regression model with 10-cross validation was used to select the optimal λ (dash line) with minimum mean square error (red dots). **D**–**F** The high-risk and low-risk groups are based on the median value of risk score (up), the survival status between high-risk and low-risk groups (middle), and the gene expression (bottom), in TCGA-PARD, GSE70768, and GSE70769 cohorts. **G**–**I** KM DFS curves of high-risk and low-risk groups in TCGA-PARD, GSE70768, and GSE70769 cohorts. **J**–**L** ROC curves for 1-, 3-, and 5-year of DFS in TCGA-PARD, GSE70768, and GSE70769 cohorts

We further investigated the prognostic values of the 76 MAGs in prostate cancer, 18 MAGs associated with DFS were identified by Univariate cox analysis (Fig. 7A). After LASSO cox regression analysis, 10 MAG signature was constructed, composed of CA14, ALDH1A2, ACSS3, ISYNA1, PYGM, TK1, HAGHL, RRM2, CD38, AK5 (Fig. 7B, C). The risk score of each sample was calculated, and then all prostate cancer patients in training (TCGA-PRAD) and testing sets (GSE70768 and GSE70769) were divided into high-risk and low-risk groups with the median value of risk score (Fig. 7D–F). The patients with higher risk scores indicated a greater number of recurrences both in the training set and two testing sets (Fig. 7D–F). The patients with high-risk scores exhibited a worse DFS (Fig. 7G–I). Moreover, the ROC curves for 1-, 3-, and 5-year DFS used to evaluate the performance of MAG signature, and the AUC for 1-, 3-, and 5-year DFS in the training set were 0.744, 0.731, 0.735 (Fig. 7J). The AUC for 1-, 3-, 5-year DFS in GSE0768 dataset were 0.668, 0.712, 0.809 (Fig. 7K). The AUC for 1-, 3-, 5-year DFS in GSE0769 dataset were 0.763, 0.802, 0.772 (Fig. 7L). ROC curves demonstrated the favorable performance of risk signature.

### Correlation of the clinical characteristics and molecular subclusters

**Fig. 8:**
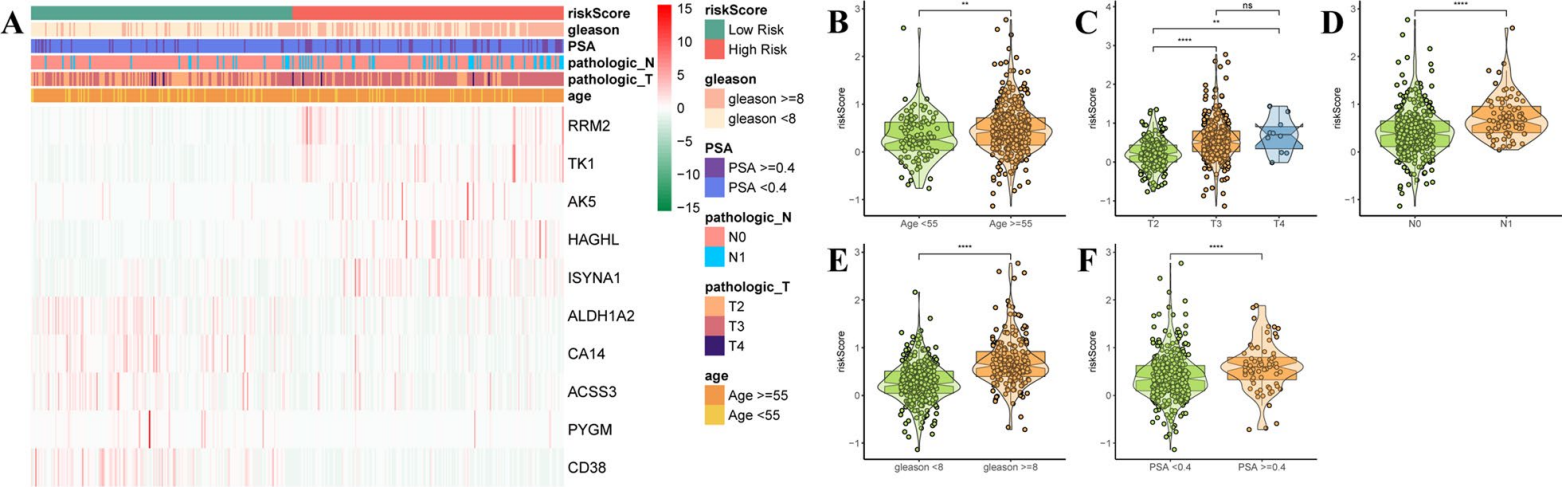
Correlation of the clinical characteristics and molecular subclusters. **A**Heatmap showed the correlation of clinicopathological characteristics (age, Gleason score, T/N stages, PSA value) and risk score in the TCGA-PARD cohort. **B**–**F** Violin plots of the different risk scores in age stratification (age < 55 and age ≥ 5), T stage stratification (T2/3/4), N stage stratification (N0/1), Gleason score stratification (Gleason score < 8 and Gleason score ≥ 8), PSA value stratification (PSA value < 0.4 and PSA value ≥  0.4)

We also investigated the correlation between risk score and clinicopathological characteristics, including age, pathological stage (T/N), Gleason score, and PSA value. As shown in Fig. 8A, risk scores were significantly associated with different ages, pathological stages, Gleason score, and PSA value. High-risk scores were associated with older patients (Fig. 8B), higher T and N stages (Fig. 8C, D), and higher Gleason scores and PSA values (Fig. 8E, F).

### Developing and validating a nomogram for prostate cancer

**Fig. 9:**
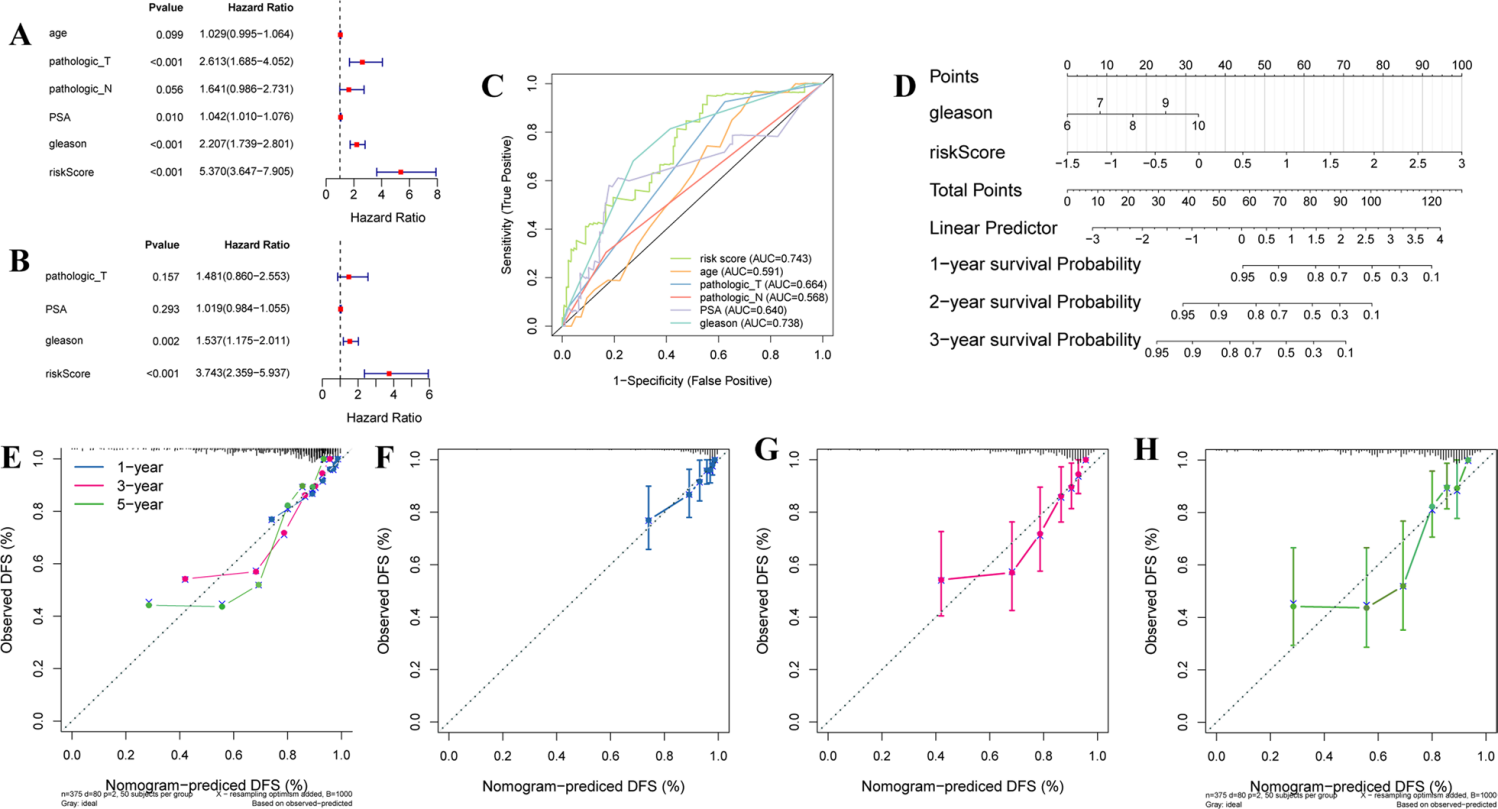
Developing and validating a nomogram for prostate cancer. **A**, **B** Forest plot of the independent factors for prognosis predicting based on univariate cox and multivariate cox analyses. **C** ROC curves validated the performance of the independent factors. **D** A nomogram for 1-, 3-, and 5-year DFS predicting based on independent factors. **E** Calibration curves for 1-, 3-, and 5-year DFS. **F**–**H** Calibration curves for 1-, 3-, and 5-year DFS, individually

Integration of the clinicopathological characteristics and risk score, univariate and multivariate cox analyses demonstrated that the risk score and Gleason score could independently predict the DFS of prostate cancer patients (Fig. 9A, B). The AUC values of risk score and Gleason score were 0.743 and 0.738, which validated the predictive ability (Fig. 9C). Based on the independent factors, we constructed a nomogram for predicting prostate cancer patients’ DFS (Fig. 9D). And the calibration curves for 1-, 3-, and 5-year DFS have confirmed the predictive accuracy of the nomogram (Fig. 9E–H).

## Discussion

In recent years, numerous studies have demonstrated that metabolic reprogramming is required for tumor initiation, malignant transformation, development, resistance to antitumor therapy (chemotherapy, radiotherapy, immunotherapy), and unfavorable outcome [37–40]. Metabolic reprogramming usually inevitably results in alterations in TME, cellular and molecular components, decreased PH value, and diverse nutritional supplements [41]. Moreover, metabolism reprogramming not only plays a crucial role in cancer signaling but also affects immune response [42]. Herein, identification of the metabolism-related subgroups of prostate cancer is a benefit for developing the treatment strategy. In this study, we identified 76 differentially expressed MAGs between prostate cancer samples and non-tumor samples. Prostate cancer patients were subsequently classified into two subgroups based on those 76 MAGs. KM curve indicated the patients in cluster 1 with poor DFS compared with those in cluster 2. These data indicated that MAGs were strongly associated with prognosis in men with prostate cancer.

Then, biological function analyses indicated that cluster 1 is mainly associated with cell cycle and metabolic pathways, such as aminoacyl tRNA biosynthesis, base excision repair, glyoxylate, and dicarboxylate metabolism, homologous recombination, mismatch repair, and protein export, and spliceosome. Previous study has demonstrated that lipid metabolism-associated pathway, including de novo lipogenesis through steroid hormone biogenesis and β-oxidation of fatty acids is related to the prognosis of prostate cancer [43]. Likewise, Berchuck et al. was found androgen signaling contributed to higher levels of lipid metabolism and effected the immune response in prostate cancer [44]. Aminoacyl tRNA biosynthesis is a hallmark of prostate cancer progression [45]. Overexpression of base excision repair related genes associated with poor survival rate for prostate cancer patients [46]. Glyoxylate and dicarboxylate metabolism served important functions in prostate cancer [47]. Moreover, gene mutations in prostate cancer involved in homologous recombination, commonly respond to PARP inhibition and platinum-based chemotherapy [48]. Mismatch repair is an important mechanism in the prevention of genetic instability, mismatch repair defects have been found in prostate cancer [49]. The spliceosome acts as a new therapeutic vulnerability in aggressive prostate cancer [50]. The above pieces of evidence have demonstrated that modulating the cell cycle and metabolism-related pathways can be used as clues for the new non-invasive early screening methods.

Meanwhile, we found cluster 2 is associated with three cardiac diseases, such as ARVC, dilated cardiomyopathy, and HCM. Although the phenomenon is rare, the similarity has been found in previous research [51]. Besides, cluster 2 is associated with two neural regulatory pathways, such as glycosphingolipid biosynthesis ganglio series and neuroactive ligand-receptor interaction. These are firstly found the unexpected phenomenon in the functional analysis of prostate cancer. Moreover, cluster 2 enriched in aldosterone-regulated sodium reabsorption, calcium signaling pathway, cytokine-cytokine receptor interaction, hematopoietic cell lineage, and vascular smooth muscle contraction. Aldosterone-regulated sodium reabsorption enriched in prostate cancer has been found in previous studies [52, 53]. And the calcium signaling pathway is found as a hallmark of aggressive prostate cancer with bone metastasis [54]. Cytokine-cytokine receptor interaction, hematopoietic cell lineage, and vascular smooth muscle contraction were firstly found in prostate cancer.

In addition, GSEA analysis also indicated that hallmarks of tumor set were enriched in cell cycle-related pathways, such as MYC targets V1 and V2, E2F targets, and G2M checkpoint in the cluster 1, and cluster 2 enriched in apical surface, apical junction, myogenesis, KRAS signaling downregulated, EMT, UV response downregulated. MYC targets V1 and V2, E2F targets, and G2M checkpoint are the four typical hallmarks of the cell cycle. MYC gene is one of the most frequently deregulated driver genes in human cancer and usually acts as a potential anticancer target [55]. E2F family not only acts as transcriptional regulators of cell cycle-dependent gene expression but also maintains genomic stability [56], upregulated E2F and E2F target in tumor link with poor prognosis in prostate cancer [57, 58]. Disruption of cell cycle checkpoints can be used as a hallmark of cancer, arresting the cell cycle by inducing the G2M checkpoints to inhibit cancer [59]. Interrupting the cell cycle may be a therapeutic strategy for prostate cancer in cluster 1. KRAS is a key oncogene in cancer, inhibition of KRAS signaling inhibits EMT in breast cancer [60]. The phenomenon indicated that cluster 2 with stromal activation. Moreover, the GSVA results supported the results, and TNFα signaling via NF-кB, inflammatory response, and IL6-JAK/STAT3 signaling were enriched in cluster 2, indicating that cluster 2 with immune activation. Similar to our results, He et al. found four metabolism-associated genes (GAS2, SLC17A4, NTM, and GC) is potential for predicting prognosis, and chemo-/immuno-therapy response in prostate cancer patients [61].

Furthermore, we investigated the TME characteristics between two subclusters. And we found cluster 1 was associated with cancer progression-relevant signaling, and cluster 2 was associated with immune activation and response-related pathways. These findings have been supported by the ESTIMATE algorithm, which was a higher ESTIMATE score, stromal score, and immune score in cluster 2 compared with cluster 1. The abundance of immune cell population in cluster 2, including B cells, CD8 T cells, cytotoxic cells, DCs, eosinophils, iDCs, macrophages, mast cells, neutrophils, NK cells, pDC, T cells, T helper cells, Tem, Tgd, Th2 cells. And Th2 and Treg cells enriched in cluster 1. There are shown significant immune heterogeneity between two metabolism-related subclusters. Whether is there any different responses for antitumor therapy between two subclusters. Firstly, we found HLA family and immune checkpoint-related genes are upregulated in cluster 2. The above results indicated that cluster 2 is associated with stromal and immune activation. And cluster 2 showed a significant response to anti-CTAL4 treatment. Moreover, cluster 1 showed more sensitivity to chemotherapy drugs, such as ABT.888, PF.4708671, GW.441756, and RO.3306. It suggested that the patients in cluster 1 are more suitable for individualized chemotherapy.

Moreover, we constructed a prognostic signature based on MAGs and divided prostate cancer patients in TCGA and two GEO datasets (GSE0768 and GSE0769) into high-risk and low-risk groups. We found higher risk scores associated with age, higher T and N stages, higher Gleason score and PSA value, and poor prognosis. The risk score and Gleason score could be used as independent factors for DFS prediction. Although MAG signature can be used as potential biomarkers for DFS prediction in prostate cancer, it is still a lack of extensive clinical validation. Gleason score remains the most reliable prognosticator in men with prostate cancer [62], nevertheless, the limitation of this method is that the Gleason grading system is based on the prostate needle biopsy, and difficult to obtain the grading of small foci of prostate cancer [63]. So, risk score and Gleason score are used as prognosticators for prostate cancer and have respective advantages and disadvantages. Therefore, maybe two prognosticators can be used together in clinical practice.

## Conclusion

Taken together, we constructed the metabolism-related molecular patterns with 79 MAGs in prostate cancer in prostate cancer with the different phenomena, which also showed significant differences in biological function, immune characteristics, and prognosis. MAGs have prognostic value in prostate cancer, and with constructed prognostic signature based on MAGs, patients were subsequently divided into two high- and low-risk score groups with different oncological outcomes. Our finding provided useful tools to manage prostate cancer. Although MAG signature has been demonstrated that revealed the reliable predictive ability for prostate cancer based on the bioinformatics analysis, the results also needed more experimental evidence to prove. Thus, the relationship of MAGs and clinicopathology parameters will verify in a larger in-house cohort and the function of these MAGs in cell phenotypes will be discussed.

## Supplementary Information

**Additional file 1. **Supplemantary file 1 NMF.

**Additional file 2. **FIGURE S1 IC50 values of 32 drugs between two subclusters.

**Additional file 3. **Table S1_TCGA-PRAD_limma_diff.

**Additional file 4. **Table S2_venn_intersectGenes.

## Data Availability

The datasets presented in this study can be found and downloaded from online databases, TCGA (https://portal.gdc.cancer.gov/), cBioPortal database (https://www.cbioportal.org/), GEO (https://www.ncbi.nlm.nih.gov/gds). Besides, the source code and processed data used for all analyses presented here can be freely obtained from https://github.com/DocotorGuo/prostate-cancer.git.
