# Supplementary figures and images for: Characterization of metabolism-associated molecular patterns in prostate cancer
Source: BMC Urol. 2023 Jun 6;23:104. doi: 10.1186/s12894-023-01275-w (PMC10243086; doi:10.1186/s12894-023-01275-w)

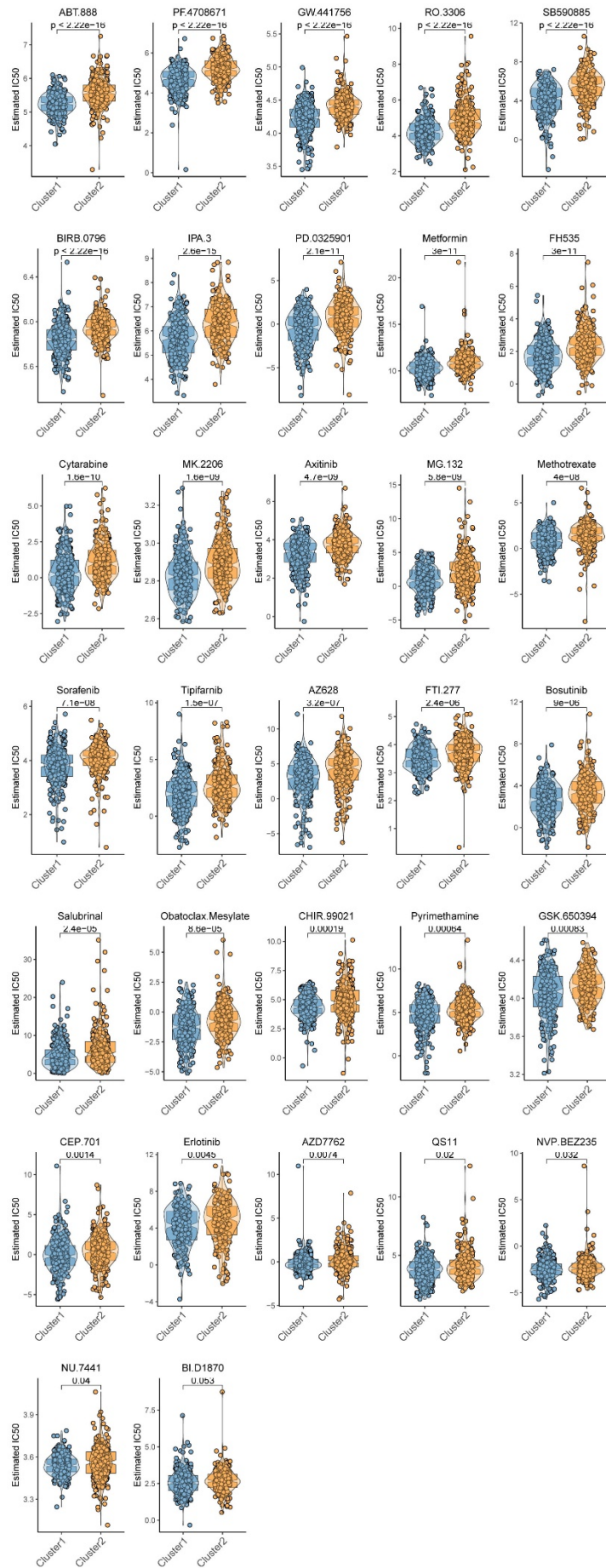

FIGURE S1 IC50 values of 32 drugs between two subclusters.

Supplement: Supplementary file 2 — Additional file 2. FIGURE S1 IC50 values of 32 drugs between two subclusters. [file 12894_2023_1275_MOESM2_ESM.pdf]
